# Effect of Structural Trimming on the Therapeutic Efficacy of K2 Capsule Depolymerase’s *Klebsiella pneumoniae* Phage B1

**DOI:** 10.3390/antibiotics15070698

**Published:** 2026-07-17

**Authors:** György Schneider, Sanjukta Patra, Karl Learmont, Péter Hampuch, Ágnes Solti-Hodován, Anita Seres-Steinbach, Marianna Horváth, Tamás Kovács, Botond Zsombor Pertics

**Affiliations:** 1 Department of Medical Microbiology, Medical School, University of Pécs, H-7624 Pécs, Hungary; hampuch.peter@edu.pte.hu (P.H.); hodovan.agnes2@pte.hu (Á.S.-H.); seres-steinbach.anita@edu.pte.hu (A.S.-S.); 2 Department of Medical Sciences and Technology, Indian Institutes of Technology, Madras 600036, Tamil Nadu, India; sanjukta@iitm.acin; 3 Department of Medical Sciences, University of Washington, Seattle, WA 98195, USA; klearm2000@uw.edu; 4 Department of Medical Biology, Medical School, University of Pécs, H-7624 Pécs, Hungary; horvath.marianna2@pte.hu; 5 Department of Biotechnology, Nanophagetherapy Center, Enviroinvest Corp., H-7632 Pécs, Hungary; t.kovacs@enviroinvest.net; 6 Plant Protection Institute, Hungarian Research Network Centre for Agricultural Research, H-1116 Budapest, Hungary; pertics.botond@atk.hun-ren.hu

**Keywords:** capsule depolymerase, *Klebsiella pneumoniae*, K2 capsule type, cloning, trimming, growth kinetics, serum resistance, mouse rescue, intraperitoneal

## Abstract

**Background/Objectives**: The treatment of multidrug-resistant and hypervirulent *Klebsiella pneumoniae* is one of today’s biggest healthcare challenges. The depletion of therapeutic options necessities the application of new methods. One promising approach is to use phage-derived enzymes that target the capsule and make the causative more visible to the immune system. As proteins, these enzymes must be optimised by reducing their size and antigenicity to make them suitable for repeated use. **Methods**: In this study, the recently isolated and cloned K2B1*orf61* K2 capsule-specific depolymerase was analysed structurally, and certain amino acid residues were deleted by cloning. The following amino acid residues were removed from the 80th (D2_N80), 115th (D3_N115), and 200th (D4_N200) N-terminal, and 250 (D5_C250) and 20 (D6_C20) from the C-terminal positions of the wild-type molecule (D1_wt). The resulting derivative molecules were compared with in vitro and in vivo tests. **Results**: In the presence of wild-type depolymerase, human serum was able to eliminate the target bacterium and save the lives of mice challenged with the bacterium in an originally lethal intraperitoneal model. However, the D5_C250 derivative lost all activity, meaning that the bacteria with the K2 capsule in the serum survived and all the mice died. Derivatives D2_N80, D3_N115, and D6_C20 exhibited prolonged activity in the serum killing assay, effectively eliminating bacteria within 5 h. Similar activity differences were revealed in the intraperitoneal experiment: D5_C250 had no rescue effect; however, D1_wt, D2_N80, D3_N115, D4_200, and D6_20 resulted in survival rates of 100%, 60%, 80%, 100%, and 20%, respectively. **Conclusions**: Our results demonstrate that molecular trimming is a promising procedure for developing ideal therapeutic depolymerases.

## 1. Introduction

*Klebsiella pneumoniae* is one of the most common pathogens, causing pneumonia, urinary tract infections, and bloodstream infections. It is also frequently associated with nosocomial infections, which can lead to sepsis [1,2]. Treating infections caused by this bacterium is becoming increasingly challenging, since strains with acquired beta-lactamases, such as ESBLs (extended-spectrum beta-lactamases) and different carbapenemases, are emerging in addition to the intrinsic AmpC lactamases present in *K. pneumoniae* [3]. The only alternative treatment option for these isolates is systemic polymyxin use, a highly toxic last-resort antibiotic [4]. The situation is further exacerbated by an increasing number of reports of severe community-acquired infections with unusual clinical presentations for *K. pneumoniae*, such as liver abscesses, endophthalmitis, and meningitis, which often have a fatal outcome. These strains are known as hypervirulent *K. pneumoniae* (hvKP) [5], and they are frequently associated with multidrug resistance (MDR) or extensively drug resistance (XDR) [6,7]. Due to its antibiotic and multidrug resistance, *K. pneumoniae* has become one of the most intractable Gram-negative bacteria [8].

The capsule is the key virulence factor of *K. pneumonia*. It facilitates adhesion and prevents phagocytosis and complement-mediated killing. This enables the bacteria to survive in the body, causing severe, invasive infections. However, the capsule can also impede access to antibiotics [9]. This is why the capsule is considered the most important target for therapeutics and vaccines [10]. Functional studies carried out in animals have demonstrated that the absence or damage of this extracellular matrix layer sensitises the *K. pneumoniae* cells, and dramatically decreases survival and pathogenicity [11]. A major challenge in developing *K. pneumoniae*-targeted therapies is the wide structural variation in capsular polysaccharide (CPS) chains. Due to these differences, the capsule is classified into over 80 serological types (K antigens) and over 140 genetically distinct capsular locus types [12]. Due to the differences in thickness and glycan structure, virulence levels are not equal between different *K. pneumoniae* capsule types [13]. Due to its resistance, K2 is one of the most prevalent serotypes that can be isolated from patients, particularly in cases of liver abscesses and endophthalmitis [14,15,16,17,18,19,20].

One strategy for eliminating *K. pneumoniae* from the human body is to damage its extracellular matrix layer, thereby exposing the bacterium to the immune system. Bacteriophage enzymes appear to be a suitable option for this task, as they can act in a capsule-type-specific manner [21]. Capsule-type-specific bacteriophages carry enzymes that specifically degrade the capsule—capsule depolymerases [22]. Recent studies have produced both disappointing and promising results. Some were successful in vitro but not in vivo [23]. Other studies have demonstrated moderate in vivo efficacy, while others have shown a synergistic effect between the applied capsule depolymerase and the state of the immune system [24,25]. In other cases, the antibacterial effect of depolymerases was inhibited in vivo. Targeted modification strategies, such as domain trimming and epitope engineering, can lead to depolymerases with improved stability and decreased antigenicity. This would enable the long-term and repeated use of therapeutic depolymerases in the human body.

We recently sequenced the lytic bacteriophage B1 (MW672037) against the K2 capsule-expressing 52145 *K. pneumoniae* strain and identified its functional capsule depolymerase (*orf61*, or KP2B1*orf61*) [26]. We also revealed that the expressed depolymerase can function as a helper enzyme, opening up the 52145 capsule and enabling phage 731 to access its receptor, which is blocked by the K2 capsule [27].

Some recent extensive in silico analyses have focused on the structural organisation of capsule depolymerases [28]. Analyses of the tripartite relationship between depolymerase specificity, capsular serotype, and phage host spectrum revealed a complex structure with distinct features among different depolymerases [25,29]. Although this has been demonstrated extensively, the aim of this study was to create a basic functional map and reveal the relationship between structure and function. With regard to their potential as therapeutic agents, it is widely accepted that capsule depolymerases are ideal candidates.

This study investigated the effects of structural trimming on the therapeutic efficacy of wild-type KP2B1*orf61* depolymerase (wild-type/C1). Five different depolymerase derivatives were constructed from the wild-type enzyme by deleting the following amino acids: D1_wt (no deletion)—wild-type K2B1*orf61*; D2_N80 (the first 80 N-terminal); D3_N115 (the first 115 N terminal); D4_N200 (the first 200 N-terminal); D5_C250 (the last 250 C-terminal); and D6_C20 (the last 20 C-terminal). The in vitro and in vivo effects of these derivatives were then compared with those of the wild-type enzyme.

## 2. Results

### 2.1. Structural Design Derivatives of K2B1orf61 Depolymerase

The project’s key enzyme was K2B1*orf61* depolymerase, whose coding region was previously identified in K2 capsule-specific bacteriophage B1. This bacteriophage was originally isolated against the *K. pneumoniae* reference strain 52145 [26]. The depolymerase’s coding gene (*orf61*) was recently cloned, expressed, and characterised in terms of its function [27]. As part of this study, we designed five different derivatives/constructs by trimming the functionally active wild-type K2B1*orf61* depolymerase. For the D2 derivative, the first 80 amino acids (N80) from the N-terminal region were deleted. For D3, the first 115 amino acids (N115) were deleted, and for D4, the first 200 amino acids (N200). Two constructs were trimmed from the C-terminal end: D5, where the last 250 amino acids (C250) were deleted, and D6, where the last 20 amino acids (C20) were deleted.

**Figure 1:**
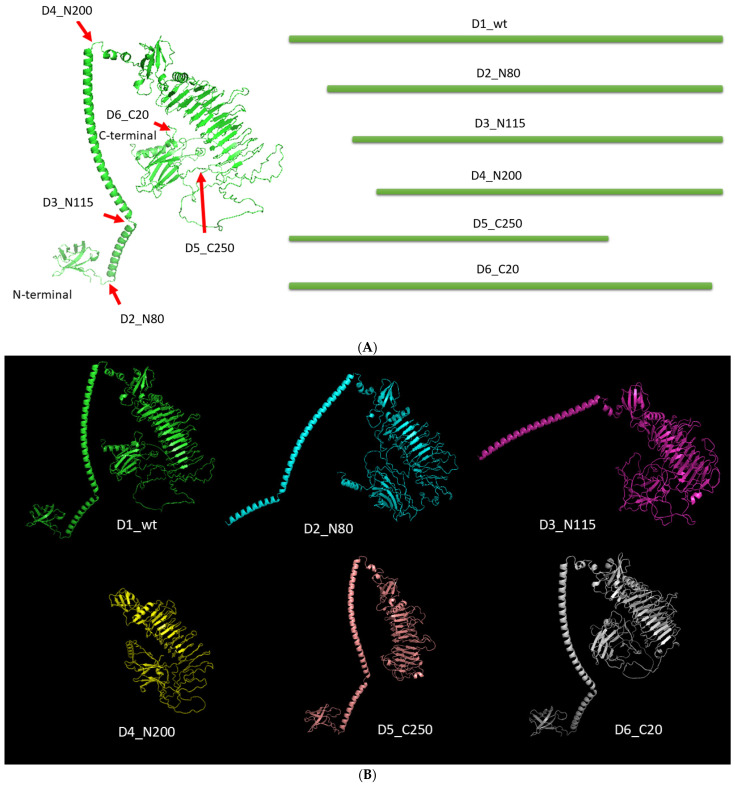

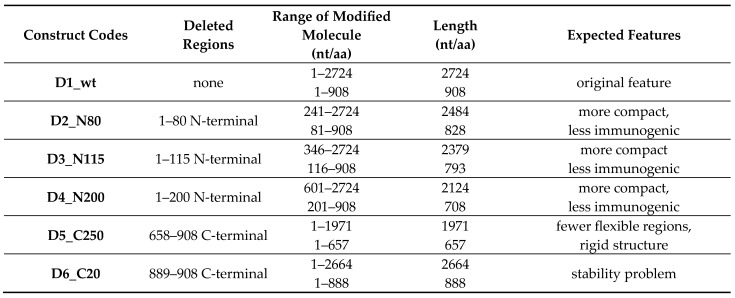
Trimming of the depolymerase K2B1*orf61* was performed based on 3D prediction. Predicted 3D structures of the wild-type depolymerase and trimming sites on the molecule and on the linearized amino acid sequence are shown (**A**). Altogether, 5 different constructs were designed and later expressed. Their predicted 3D structures are presented (**B**). The table below shows the deleted regions, intact parts, and lengths. Construct D1 is the original wild-type depolymerase, while constructs D2 to D6 are trimmed derivatives of K2B1*orf61*. More detailed 3D images are available in Supplementary Data S1 and can be visualised in PyMOL or other appropriate software.

Three-dimensional structural images of the wild-type enzyme revealed that the original K2B1*orf61* depolymerase consists of two main parts (Figure 1). At the N-terminal end, there is a β-helix-rich structure consisting of 200 amino acids, which begins with a globular structure consisting of around 80 amino acids. A long linear linkage connects this globular structure to the other large part of the enzyme, (from amino acid 200 onwards) which can be divided into at least four additional parts. A linker-like region connects the aforementioned long β-helix-rich structure to the largest mass of the enzyme. This mass is connected to a smaller region and ends in a small β-helix structure, which forms a small arm at the end of the molecule (Figure 1).

### 2.2. Cloning and Expression of Depolymerase Derivatives

Sequencing the cloned depolymerase derivatives confirmed that the PCR fragments were successfully cloned and that no frameshifts occurred during the planning and construction process. The cloned proteins were successfully expressed in *E. coli* KRX cells. Following purification, each trimmed molecule ran as a single band on 1D PAGE, with sizes varying between 70 and 100 kDa, depending on the derivative. The enzyme derivative concentrations were adjusted to 2 mg/mL in a PBS buffer. Activity testing on the lawn of the KL2 *K. pneumoniae* strain 52145 revealed that all derivatives were active. While the activities of D2, D3, and D4 were comparable to the wild type, drastic activity loss was observed in the case of D5, with minimal loss observed in the case of D6. Notably, despite the absence of 250 amino acids in the D5 sequence, this derivative exhibited a modest clearing effect on the strain 52145 lawn.

### 2.3. Serum Killing Assay

Since the K2 capsule and the O1 antigen are both important influencing factors of the complement system, a serum killing assay was performed. The complement system is only able to kill bacteria if the membrane attack complex can be formed on the surface of a bacterium cell. Therefore, both the K2 and O1 structures impede its action; that is why the most ideal construct for us to detect the activity of K2 capsule depolymerase was the 52145 Kp mutant, which lacked O1 but had the K2 on its surface. However, in the tests, all of the mutants were screened.

The *K. pneumoniae* wild-type strain 52145 and its capsule mutants, 52145 O1:K- and 52145 O-:K2, are serum-resistant. However, the double mutant strain, 52145 O-:K-, is serum-sensitive when investigated over a 24-h period in the presence of 50% human serum. The sensitivity of strain 52145 O-:K- is striking, with the number of cells dropping by approximately two orders of magnitude within the first hour of incubation. If the bacterium possessed the O1 LPS, the K2 capsule, or both, then it survived and proliferated in the presence of serum. Conversely, the survival and proliferation of all four strains remained unaffected by heat-inactivated (56 °C for 30 min) serum.

**Figure 2:**
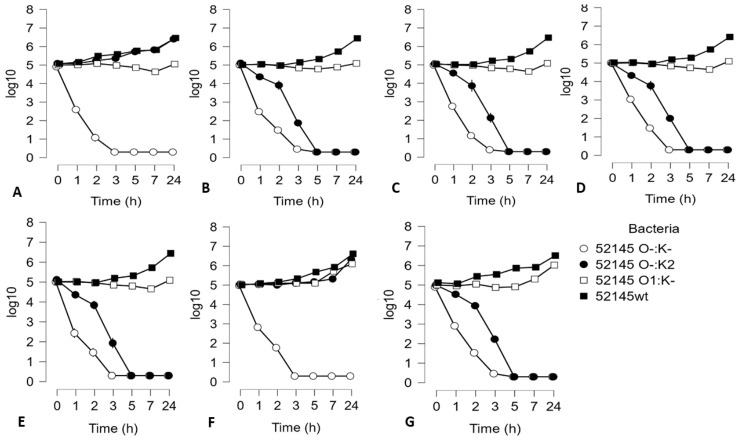
The time-dependent bactericidal effects of the depolymerase derivatives in 50% human serum, tested on the survival of the wild-type 52145 strain, the K2 mutant 52145 O1:K-, the LPS mutant 52145 O-:K2, and the double mutant 52145 O-:K- strains. The depicted effects were observed in the test tubes containing only the serum (**A**), the wild-type depolymerase K2B1*orf61* (**B**), derivatives lacking (**C**) the first 80 amino acids at the N-terminus, (**D**) the first 115 amino acids at N-terminus, (**E**) the first 200 amino acids at the N-terminus, and derivatives lacking (**F**) derivatives lacking the last 250 amino acids at the C-terminus and (**G**) the last 20 amino acids at the C-terminus.

Regarding the co-incubations with depolymerase treatments at a concentration of 100 μg/mL, the wild-type depolymerase K2B1*orf61* and its derivatives lacking the first 80 (D2), 115 (D3), 200 (D4), or last 20 (D6) amino acids were able to sensitise the 52145 O-:K2 *K. pneumoniae* strain (Figure 2). This was the only strain on which these depolymerases acted. While their effects were slightly slower, the number of living bacterial cells dropped to around zero by the end of the fifth hour of co-incubation in each case. Conversely, the depolymerase derivative (D5), which lacked 250 amino acids from the C-terminal region of the enzyme, was ineffective, and no decrease in the number of bacteria present could be observed. The results showed that the bacterial cell numbers could be influenced by applying depolymerase treatments, with either O1 LPS or the K2 capsule containing derivatives alone. Preliminary testing was also carried out in heat-inactivated serum, in which all the strains exhibited robust viability.

When we examined whether there were significant differences between the individual bacterial strains at each time point, we obtained the following results: At time point 0, there was no significant difference between 52145 O-:K- and the bacterial type present at the other time point (p_(bonf) = 1.000). As the incubation time increased, differences among the bacterial types under investigation became apparent at each time point. At 1, 2, 5, 7, and 24 h, O:K- showed significant differences compared to all the other types (p_(bonf) < 0.001). The same was true for O-:K2 at 1, 2, 5, 7, and 24 h. However, there was no significant difference between O1:K- and WT during the first hour (p_(bonf) = 1.000). Furthermore, O1:K- still did not show a significant difference from WT in the second hour; however, this time, the p-value was p = 0.080. From the third hour onwards, O1:K- showed a significant difference from WT throughout the remainder of the experiment (p_(bonf) < 0.001).

When we examined the significant correlation between serum levels and time, we found that there was no significant difference between the groups at 0 h (p_(bonf) = 1.000). However, after one hour of incubation, significant differences were observed (p_(bonf) < 0.001) between the control serum and D1_wt (p_(bonf) = 0.008), between the control serum and D4_N200 (p_(bonf) < 0.001), between D1_wt and D5_C250, and between D4_N200 and D5_C250. At the second, third, fifth and seventh hours, there was no significant difference between the control serum and D5_C250 alone. However, significant differences were observed when compared with the other sera tested. Significant differences were also evident between D1_wt and D5_C250; D2_N80 and D5_C250; D3_N115 and D5_C250; and D4_N200 and D5_C250 (p_(bonf) < 0.001) during these hours. Additionally, significant differences were observed in the seventh hour between D4_N200 and D6_C20 (p_(bonf) < 0.043) and between D5_C250 and D6_C20 (p_(bonf) < 0.001).

By the 24th hour, a significant difference was observed between D5_C250 and the control serum (*p* < 0.001). Furthermore, significant differences were observed between D1_wt and both D5_C250 (*p* < 0.001) and D6_C20 (*p* = 0.001). Additionally, significant differences were observed between D2_N80 and D5_C250 (*p* < 0.001), as well as between D2_N80 and D6_C20 (*p* = 0.003). D3_N115 also showed a significant difference compared to both D5_C250 and D6_C20 (*p* < 0.001). Additionally, significant differences were observed between D4_N200 and D5_C250 (*p* < 0.001) and between D4_N200 and D6_C20 (*p* = 0.002). A significant difference was also detected between D5_C250 and D6_C20 (p_(bonf) < 0.001). In summary, the statistical analysis suggests that the K2 capsule plays an important role in strain 52145’s resistance to whey, but also that the O-antigen plays an important role in early phase defence.

### 2.4. Bacterial Growth Kinetics in the Presence of Depolymerases

**Figure 3:**
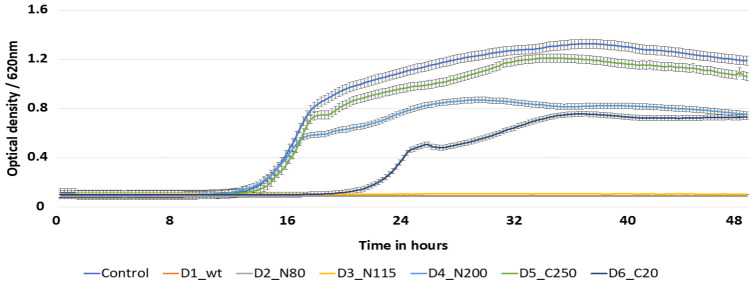
The bactericidal efficacies of the K2B1*orf61* wild-type depolymerase (D1) and its five derivatives (D2–D6), tested in the presence of 50% human serum. The K2 capsule-expressing O1 mutant derivative of 52145 (O:K2), which is serum-resistant but becomes serum-sensitive if it loses its capsule in the presence of an active capsule depolymerase, was used for the experiment. The initial CFU count of the bacterium was 10^2^ CFU/mL, and proliferation was monitored based on changes in optical density at 620 nm, for 24 h.

To evaluate the impact of the depolymerase derivatives on bacterial growth, the strain 52145 O-:K2 of *K. pneumoniae* (control) was used. After approximately 16 h of incubation at 37 °C, this sample reached a maximum OD_620_ value of 1.4 (CFU 10^6^/mL) by the 32nd hour of incubation, with an initial CFU of 10^2^ CFU/mL (Figure 3). When this strain of bacterium was co-incubated with purified K2B1*orf61* wild-type (D1_wt) depolymerase at a concentration of 100 μg/mL, the bacteria present in the 96-well plate at a concentration of 10^3^ CFU/mL were unable to survive. This resulted in no growth in turbidity in the LB medium in the wells. The results showed that D2_N80 and D3_N115 were able to hinder the proliferation of 52145 O-:K2, demonstrating activity similar to that of the wild-type K2B1*orf61* depolymerase. Growth of the test bacterium in the presence of the D4_N200 depolymerase derivative was evident, albeit less intense than in the control group. Growth was even more subdued when the D6_C20 derivative of the wild-type depolymerase was present in the bacterial suspension. D5_C250 was the least effective depolymerase derivative, as it was unable to restrain the proliferation of the test bacterium 52145 O-:K2.

For statistical analysis, we calculated *p*-values using the General Linear Model (Repeated Measures) in SPSS Statistics, version 26. According to the ‘SPSS Tests of Within-Subjects Effects’ table, *p* < 0.001. This shows that the kinetic values changed significantly over time. We assessed the differences between groups using the Pairwise Comparisons table with the Bonferroni correction. There were significant differences between our different treatment groups (Control, D1_wt, D2_N80, etc.) throughout the entire time course (*p* < 0.05).

### 2.5. In Vivo Rescue Comparison Ability of the Depolymerases in Mice

**Figure 4:**
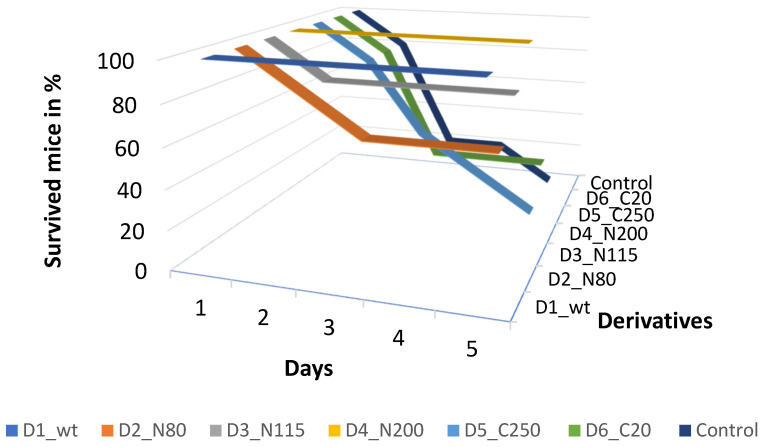
Demonstration of the in vivo efficacy differences between the depolymerase derivatives and the wild-type K2B1orf61 (wt). Survival rates of the 52145 O-:K2-challenged mice groups are depicted. Each group contained 5 mice, and the cumulative results of the survival of these 5 mice are also shown. After treatment, animals were monitored for five days.

To demonstrate the differences in in vivo efficacy between the expressed depolymerases, the depolymerase derivatives were administered intraperitoneally to mice challenged with 52145 O-:K2. In the control group, only sterile PBS was administered after infection; by the second day, the animals were showing signs of systemic infection, such as lethargy and ruffled fur. All five control group mice died by the end of the fourth day. However, when a 100 μg wt depolymerase K2B1*orf61* suspension was administered intraperitoneally one hour after the bacterial challenge, all the bacteria survived (Figure 4). The derivative D4_N200 exhibited a comparable protective effect: all five mice in this group survived until the end of the fifth day. At this point, the animals showed no signs of infection, such as lethargy or ruffled fur. The least effective depolymerase derivatives were D5_C250 and D6_C20. Based on our results, these derivatives had no protective effect when applied in a large dose (100 μg/mouse). The effects of the other two depolymerase derivatives, D2_N80 and D3_N115, were somewhat contradictory: they exhibited some protective effects, but there were differences between the two groups. More precisely, D3_N115 appeared to provide slightly greater protection than D2_N80. In an experiment where the bacterium used for the challenge killed the mice within five days, the survival rates were 100%, 60%, 80%, 100%, 0%, and 20% for D1_wt, D2_N80, D3_N115, D4_N200, D5_C250, and D6_C20, respectively (Figure 4).

To analyse mouse survival, we used the generalized linear model within the JASP programme, assuming a Poisson distribution. Based on the analysis, *p* = 0.018, indicating a significant difference in the combined effect of days and serum types, which influence survival. Upon further examination of the results, we observe that D1_wt (*p* = 0.023) and D4_N200 (*p* = 0.023) have positive estimate values (0.821), meaning that the survival rate in these groups was significantly higher than in the control group. 

When we examine the days, we see that significant differences can be observed in our results on days 4 and 5 (*p* < 0.005); however, based on the estimate values, these figures become increasingly negative as time progresses—that is, the further forward we go in time, the higher the mortality rate becomes among the mice.

## 3. Discussion

Bacteriophage-derived capsule-degrading enzymes (capsule depolymerases) are potent therapeutic agents for treating multidrug-resistant and hypervirulent *Klebsiella pneumoniae* infections. While several studies have reported on their in vivo efficacy, few have addressed the fact that these enzymes are proteins, and repeated administration can reduce their efficacy. One way to address this issue is to reduce antigenicity by either decreasing the size of the enzymes or replacing crucial immunogenic amino acids with more neutral ones. In this study, we initially focused on size reduction by further analysing the recently identified, expressed, and partially characterised K2 capsule-type-specific depolymerase, K2B1*orf61* [26,27].

The discovery of a relatively distinct 200-amino-acid-long linear segment within the enzyme indicated that this N-terminal region might be involved in the binding to the phage filament or basal plate. This is why we aimed to cut down this long sequence from the rest of the enzyme. However, no matter how distinct it seemed, we could not forget that different proteins can be affected in terms of stability and function by relative distinct regions. Therefore, in addition to N200 (D4), we also planned derivatives of D2 and D3, in which only the globular N-terminal end was removed from the slightly broken parts of the molecular structure, at position N115. As the other major part of K2B1*orf61* consists of one large and one small globular unit, as well as a short lever-like structure consisting of a short beta-chain at the C-terminal end, we assessed their importance in terms of efficacy by trimming them. The D5_C250 derivative lacked the small globular unit and the short beta-chain linkage, while the D6_C20 derivative lacked the unit of nearly 20 amino acids from the C-terminal end.

Recent studies have reported that the poor in vitro efficacy of expressed depolymerases could be due to the purified enzyme failing to form the expected trimer found in bacteriophages [25]. This study confirmed that excessive polymerisation hindered the enzyme’s precise function. The authors revealed that the 18 depolymerase molecules stuck together, thereby inhibiting function. However, as in other studies, we did not investigate the polymerisation rate of the expressed depolymerases and their derivatives, since the firm functional activity of K2B1*orf61* made this unnecessary.

Even at high germ counts, regular sampling of serum samples revealed that the wild-type depolymerase could eliminate strain 52145 O-:K2 from the test wells by enabling the complement system to bind to the bacterium’s cell surface. This cell surface lacked O1 LPS, an inhibitory LPS type for a complementary effect. This effect clearly occurred due to the complement system, since the growth of bacteria in heat-inactivated serum resembled that of the wild-type control. The decreased serum resistance of the simple capsule mutant 52145-∆wcaK2 (O1:K-) showed that the capsule plays a significant role in this bacterium’s serum resistance. However, comparing this result with that of the double mutant 52145-∆wcaK2∆waaL (O-:K-) confirmed the previous observation that the O1 LPS also plays a crucial role in serum protection [30]. This is why we based our experimental setup on the 52145 O-:K2 mutant. This allowed us to unambiguously detect the capsule-degrading effect and differences in the efficacies of the constructed depolymerase derivatives. It is also worth mentioning that the O1 and O2 LPS are both responsible for serum resistance in *K. pneumoniae*, and these O serotypes occur additively in 55–65% of isolates [31,32,33]. Therefore, future studies should focus on constructing hybrid enzyme complexes that are effective against O1, O2, and certain capsule types. Recently, some studies have focused on combining depolymerases to create hybrid enzymatic complexes [22,34,35]. A crucial aspect of this, however, will be to decrease antigenicity in order to ensure the permanent systemic use of a phage complex in the human body. The two most important issues to be solved are trimming and reducing antigenicity.

The results of the in vivo efficacy testing in mice were promising, clearly depicting that K2B1*orf61* and some of its derivatives were effective in the body. For this purpose, we used the intraperitoneal mouse model. This is an accepted model for testing *K. pneumoniae* virulence, though intravenous, lung, and nostril colonisation infection models are also accepted [36]. The infection course in this model was appropriate for the 52145 O-:K2 strain. Some recent studies have reported that hypervirulent *K. pneumoniae* isolates can kill challenged mice within two days, and such models are useful for testing therapeutic efficacy in later development stages [37]. Nevertheless, the ‘four-day infection pattern’ effectively demonstrated the differences in efficacy between the tested depolymerase enzymes and their derivatives. As in other studies, we demonstrated that expressed and purified depolymerase is an effective agent in vitro in the presence of human serum and in vivo in bacterium-challenged mice [38].

## 4. Materials and Methods

### 4.1. Bacterium Strains and Media

This study examined the wild-type (wt) strain of the human isolate *Klebsiella pneumoniae* 52145 and its corresponding isogenic mutant strains (Table 1). The bacteria were routinely cultured on Luria–Bertani (LB) agar plates or in LB medium at 37 °C with shaking at 120 rpm. To prepare a bacterial lawn, 100 µL of the cultures grown overnight in liquid medium were spread onto solid LB agar plates and incubated overnight at 37 °C. To propagate the bacteria, cultures were grown overnight in a liquid medium at 37 °C using an orbital shaker set to 120 rpm.

### 4.2. Design of Derivatives

The depolymerase gene K2B1*orf61*, which was previously cloned, was considered as the original depolymerase in this study. This construct was referred to as construct one (D1) or a wild type (wt). Five different constructs (D2–D6) were designed by deleting characteristic regions of this depolymerase (Figure 1). The most important aspect of this study was to identify characteristic structural regions and delete them and to obtain feedback about their importance concerning function.

In derivative D2, the first 80 amino acids (N80) from the N-terminus were deleted. In D3, 115 amino acids (N115) were deleted; in D4, 200 amino acids were deleted from the N-terminus. In constructs 5 and 6 (D5 and D6), 250 and 20 amino acids containing C-terminal regions were deleted from the 908 amino-acid-long original or wild-type molecule. Before cloning, the planned structures of the trimmed constructs were visualised, as follows. Manual trimming of the nucleotide sequences was carried out in Excel (2021) using the MID command. These trimmed nucleotide sequences were then converted into proteins using the DNA-to-amino acid converter tool from CUSABIO (https://www.cusabio.com/, accessed on 11 May 2026) at the CUBIO Innovation Center (Houston, USA). The resulting sequences were submitted to AlphaFold2 Colab (https://colab.research.google.com/, accessed on 11 May 2026), where the structural predictions and visualisations were carried out. Predictions were also performed using the RoseTTAFold platform (Washington, USA) in Colab (https://colab.research.google.com/github/sokrypton/ColabFold/blob/main/RoseTTAFold2.ipynb, accessed on 11 May 2026) (Google LLC, Mountain View, CA, USA). A 3D visualisation of the derivatives can be performed by using PyMOL v.2.6.2 (Schrödinger Inc., New York, NY, USA) with the generated files (Supplementary Material Data S1).

### 4.3. Cloning the Structural Derivatives of K2B1orf61

Cloning of the depolymerase gene (*orf61*) of *K. pneumoniae* phage B1 into the pRSET A expression vector (Thermo Fisher Scientific, Waltham, MA, USA) has recently been described. The complete gene, which contains the ATG START and TAA STOP codons, is 2724 bp long and encodes 908 amino acids.

Each construct was originally cloned using the primers listed in Table 2. Phusion High-Fidelity DNA Polymerase (New England Biolabs, Ipswich, MA, USA) was used for the PCR amplification procedures, following the manufacturer’s instructions. The reaction mixture consisted of 200 µM of each dNTP, 0.5 µM of a forward primer and 0.5 µM of a reverse primer; 2 ng/µL of phage DNA; 3% (*v*/*v*) of DMSO; and 0.02 U/µL of Phusion DNA polymerase in 1× Phusion HF buffer. The total reaction volume was 20 µL.

PCR cycling started with an initial denaturation step at 98 °C for 1 min, followed by 30 amplification cycles consisting of denaturation at 98 °C for 20 s, primer annealing at 60 °C for 15 s, and extension at 72 °C for 3 min. A final extension step was then performed at 72 °C for 5 min.

The amplified products were separated by agarose gel electrophoresis using a 0.8% agarose gel prepared in a 1× TAE buffer. The appropriate DNA bands were excised and purified using a NucleoSpin Gel and PCR Clean-up kit (Qiagen, Hamburg, Germany), according to the manufacturer’s instructions.

The purified PCR fragments were double-digested using BamHI and XhoI (New England Biolabs, Ipswich, MA, USA) for two hours. The enzymes were heat-inactivated at 85 °C for 15 min, and the fragments were ligated directly into the previously linearised vector (BamHI and XhoI).

The pRSETA expression vector, which was previously used in our studies to support the expression of the K2B1*orf61* depolymerase gene in the KRX background, was used for cloning purposes. This vector contains a T7 promoter, a ribosome binding site (RBS), an ATG START codon, a His tag, and an Xpress tag. A multicloning site was located downstream of these units, and the *Bam*HI and *Xho*I sites were used. A T7 terminator, in frame with the Xpress tag, was located downstream of the cloned insert. During construction, the ATG START codons were deleted from the original sequence, as the pRSET A vector contains them. However, STOP codons were added, as our construct did not contain a C-terminal tag. Sequence analysis of the clones confirmed that the constructs were correct and that we had indeed cloned the selected regions of the original K2B1*orf61* depolymerase gene without introducing any mutations or frameshifts.

The ligation mixture contained 1 µL of T4 DNA ligase, 2 µL of T4 DNA ligase reaction buffer (NEB), 10–17 µL of purified DNA, and 1 µL of linearised plasmid. The ligation reaction was incubated at 23 °C for 2 h, followed by enzyme inactivation at 65 °C for 10 min.

In the initial trials, the circularised plasmids were introduced into electrocompetent *E. coli* KRX cells (Promega, Vienna, Austria) using heat shock transformation (43 °C for 90 s). If difficulties arose, electroporation (1 mm cuvettes, 1.8 kV, 600 Ω) using the Gene Pulser Xcell Electroporation System (Bio-Rad, Hercules, CA, USA) was carried out. The cells were then revitalised in non-selective LB medium at 37 °C for 1 h and plated onto LB medium plates containing 100 µg/mL ampicillin. After plating, the cells were incubated overnight at 37 °C. Resistant clones were then retrieved and inoculated in 5 mL of LB medium supplemented with 100 µg/mL ampicillin. The culture was grown overnight at 37 °C, and glycerol stocks were prepared by mixing 500 µL of the culture with 500 µL of 50% glycerol. Proper cloning was confirmed by plasmid isolation followed by DNA sequencing.

### 4.4. Expression and Purification of the Modified K2 Depolymerases, Drop Plate

Fresh cultures were inoculated with overnight cultures (5 mL) of KRX and KRX, harbouring the *orf61*-pRSET A plasmid construct, and they were grown to the logarithmic phase (OD600 = 0.8). Protein expression was induced by adding isopropyl-β-D-thiogalactoside (IPTG) to achieve a final concentration of 2 mM. To evaluate expression efficiency, 1 mL samples were collected at 2, 4, and 6 h after induction.

The samples were then centrifuged, and the resulting pellets were either resuspended in distilled water and boiled at 100 °C for 10 min or resuspended in sonication buffer (pH 7.4, 50 mM Tris, and 1 mM EDTA) for sonication-based cell disruption. Sonication was performed in four cycles of pulsed operation (0.5 s on, 0.5 s off) for one minute at an amplitude of 25–30%. Between cycles, the samples were incubated on ice for 1–2 min.

The boiled or sonicated samples were then mixed with 5× sample buffer at a ratio of 1:4 (*v*/*v*). This contained 0.6 mL of 1 M Tris (pH 6.8), 5 mL of 50% glycerol, 2 mL of 10% sodium dodecyl sulfate (SDS), 0.5 mL of β-mercaptoethanol, bromophenol blue, and sufficient distilled water to obtain a final volume of 10 mL. The samples were separated by electrophoresis on a 10% polyacrylamide gel for one and a half hours at 120 V using a Bio-Rad Mini Protean II system (Bio-Rad Laboratories Inc, Hercules, CA, USA). Protein bands were visualised via staining with Coomassie Brilliant Blue.

The expressed depolymerase was affinity purified using a polyhistidine tag from a 5 mL culture in the logarithmic phase, induced with 2 mM IPTG for 5 h. After centrifugation, the pellet was resuspended in His-binding buffer and sonicated as described above. Protein purification was performed using a His-Spin Protein Miniprep kit, following the manufacturer’s instructions. The purified protein was analysed by polyacrylamide gel electrophoresis, after which its activity and host range were tested.

The classical drop plate method was used to pre-screen the crude extracts of the expressed wild-type and recombinant depolymerases and to test the column-purified depolymerases, as previously described. Briefly, a bacterial lawn was formed on solid LB agar plates by spreading 100 µL of suspensions from overnight liquid cultures of *K. pneumoniae* isolates. The lawns were pre-incubated for 10–12 h at 37 °C to allow the bacteria to form a firm lawn and to allow the capsule to become visible. Then, 10 μL of the depolymerase suspension was dropped onto the lawns, and the effects were monitored every ten minutes.

### 4.5. Serum Killing Assay

To test the activity of the K2B1*orf61* depolymerase and its trimmed derivatives in human serum, the following experimental setup was performed. The tests involved the following strains of K. pneumonia: the wild-type 52145 O1:K2; the isogenic mutant lacking the K2 capsule (52145 O1:K-); the double mutant lacking both capsule and LPS (52145 O-:K-); and the O1 LPS mutant *K. pneumoniae* strain 52145 O-:K2 (Table 1). Pooled normal human serum (NHS) was collected from eight healthy volunteers. The serum resistance and sensitivities of the test strains were previously determined as described, but with some modifications. Briefly, 250 μL of a log-phase bacterial suspension (at a concentration of 10^6^ CFU/mL) was mixed with an equal volume of NHS and incubated at 37 °C for 24 h. At 0, 1, 2, 3, 5, 7, and 24 h, 20 μL samples were taken, serially diluted, and plated for colony counting. As part of the preliminary experiment, the survival and viability of the bacteria were also tested in heat-inactivated NHS (HI-NHS). Prior to the experiments, the pooled NHS was incubated at 56 °C for 30 min to deactivate complement activity, and the tests were performed in 50% HI-NHS.

The same procedure was used for the depolymerase activity testing. The only difference was that testing was performed in the presence of K2B1*orf61* depolymerase and its derivatives (D2–D6) in separate parallel tubes. The depolymerases were added to the tubes at an end concentration of 100 μg/mL. At the relevant time points, samples were taken, and the number of living bacteria was determined by dilution and spreading on LB agar plates.

The assay was carried out in triplicates and the gained CFU values were averaged; the averaged values were plotted on a time scale.

We performed a comparative analysis of the kinetics using SPSS Statistics 26. We assessed the level of significance using the repeated measures module within the general linear model. We considered a *p*-value of less than 0.005 to be significant.

### 4.6. Efficacy of the Depolymerase Derivatives on Bacterial Growth Kinetics

The following experiment was conducted to determine the bactericidal activities of the depolymerase derivatives in the presence of 50% pooled human serum. This procedure is generally similar to the serum resistance test described above, but with some key differences. For the test, a six-hour logarithmic phase culture of the serum-resistant *K. pneumoniae* strain 52145 O-:K2 was used, which was originally started from a 14 h ON culture. The bacteria were diluted to a concentration of 10^3^ CFU/mL, and 100 μL of this solution was pipetted into the reaction wells. An additional 100 μL of serum was then pipetted onto this. The 200 μL volume consisted of 5 μL of PBS being added to the bacterium control wells; 5 μL of K2B1*orf61* depolymerase being added to the three depolymerase control wells (D1); and 5 μL of depolymerase derivatives (D2–D6), prepared from 2 mg/mL stock solutions, being added to the wells to monitor the derivatives’ activity.

Measurements were carried out using a multimode reader (Allsheng, Hangzhou, China) over a 24 h period. Optical densities were recorded every 15 min at a wavelength of 620 nm. Prior to each measurement, the samples were shaken for five seconds. The collected data were exported to Excel and used to generate growth curves. All measurements were performed in triplicate.

### 4.7. In Vivo Rescue Comparison in a Mouse Model

The *K. pneumoniae* strain 52145-∆waaL, which lacks the O1 lipopolysaccharide (LPS) but expresses the K2 capsule, was used in an intraperitoneal mouse model to reveal differences in in vivo efficacy among the depolymerase derivatives constructed in this study.

The experiments were performed with 8–10-week-old black female BL67 mice (C57/BL67, weight: 18–25 g, origin: Charles River Laboratories). The animals were housed in a room with a standard temperature of 23 °C ± 1 °C and 12 h light/dark cycles for the bacterial challenge. The 52145-∆waaL strain grew to an OD_620_ nm of approximately 0.5 in 5 mL of LB broth at 37 °C with shaking at 120 rpm. The bacterial suspension in the log phase was then centrifuged for 1 min at 12,000 rpm, washed with PBS, and adjusted to an OD_620_ nm of 1.0. This suspension was diluted 100-fold, and 250 μL doses were administered to the experimental animals via the left abdominal intraperitoneum without anaesthesia using a G10 hollow needle. One hour after the bacterial challenge, 250 μL doses were administered into the right abdominal intraperitoneum of the animals according to the following distribution. The animals were divided into seven groups, each containing five mice. All animals received the 52145-∆waaL strain at the aforementioned dose. Members of the first group (G1) received 100 μg of the wild-type depolymerase K2B1*orf61* (D1_wt). Similarly, members of groups 2–6 received the depolymerase derivatives D2_N80, D3_N115, D4_N200, D5_C250, and D6_C20, respectively. The animals in group 7 served as the control group and received only PBS. All groups were monitored daily for seven days before euthanasia.

All mouse experiments were conducted in accordance with the European Federation for Laboratory Animal Science Associations’ guidelines. The Animal Welfare Committee approved all protocols and procedures involving Enviroinvest Co.’s animals (Permit Number: BAI/35/867 6/2019; approved on 16 May 2019).

## 5. Conclusions

In this study, we demonstrated that reducing the size of depolymerases is an effective approach to developing therapeutic agents. This produces a more compact, yet still active, form available, as demonstrated with D4_N200. This form is an ideal candidate for further targeted manipulations of antigenic features. The mapping and identification of some hot spots on the molecule are underway. Another approach involves developing chimeras by fusing the engineered capsule depolymerase with the O1 and O2 LPS-degrading enzymes associated with serum resistance to *K. pneumoniae*.

## Figures and Tables

**Table 1:** Bacterium strains used in this study.

Bacterium Strain	Serotype	Reference
52145	O1:K2	[26]
52145-∆wca_K2_	O1:K-	[26]
52145-∆wca_K2_∆waaL	O-:K-	[26]
52145-∆waaL	O-:K2	own isolate

**Table 2:** The primers used in this study were employed to construct derivatives of K2B1*orf61*. The restriction enzyme in the primers is labelled in bold, while the integrated STOP codon is labelled in italics. ‘aaaa’ overhangs were used at the 5′ ends of the primers to facilitate cutting with restriction enzymes during cloning.

No.	Name	Sequence	Used for Construct
1	241Fw_BamHI	aaaa**GGATCC**CTTACCGTTGACGGACTGGC	D2
2	2724Re_XhoI_Sp	aaaa**CTCGAG***TTA*GCTACTCATAAATCCATTTGCTTTC	D1, D2, D3, D4
3	241Fw_BamHI	aaaa**GGATCC**CTTACCGTTGACGGACTGGC	D2
4	346Fw_BamHI	aaaa**GGATCC**TTCGAATCGCTGCAAAATCTTGCTAA	D3
5	601Fw_BamHI	aaaa**GGATCC**GGTCGCGTCAGTTCATTTGCTG	D4
6	2604Re_XhoI_SP	aaaa**CTCGAG***TTA*ACCAACTCTAAATCTATCAATAGCTACC	D5, D6
7	1971Re_XhoI_Sp	aaaa**CTCGAG***TTA*AAATGCGACACAGTTTATCCCAAGC	D5
8	2664Re_XhoI_Sp	aaaa**CTCGAG***TTA*ATTGGCACTGTTAACAAATCCATATCTTATT	D6

## Data Availability

The original contributions presented in this study are included in the article/Supplementary Materials. Further inquiries can be directed to the corresponding author.

## Supplementary Materials

The following supporting information can be downloaded at: https://www.mdpi.com/article/10.3390/antibiotics15070698/s1, Supplementary Material Data S1: PDB three-dimensional files of the wt and derivative molecules (visualization with PyMOL or relevant software).

## Abbreviations

The following abbreviations are used in this manuscript:

*K. pneumoniae*	*Klebsiella pneumoniae*
MDR	multidrug resistant
NHS	normal human serum
PAGE	polyacrylamide gelelectrophoresis
wt	wild type
